# The Relationship Between Preoperative Vitamin D Levels and Keloid Recurrence

**DOI:** 10.1111/jocd.16687

**Published:** 2024-11-28

**Authors:** Da Woon Lee, Daihun Kang, Boram Ha, Da Hye Kim, Choong Hyun Chang, Tae Hwan Park

**Affiliations:** ^1^ Department of Plastic and Reconstructive Surgery, College of Medicine Soonchunhyang University Cheonan Republic of Korea; ^2^ Department of Plastic and Reconstructive Surgery Ewha Womans University Seoul Hospital Seoul Republic of Korea; ^3^ Department of Radiation Oncology, Dongtan Sacred Heart Hospital Hallym University College of Medicine Hwaseong Republic of Korea; ^4^ Department of Plastic and Reconstructive Surgery, Dongtan Sacred Heart Hospital Hallym University College of Medicine Hwaseong Republic of Korea; ^5^ Hallym University College of Medicine, Chuncheon‐si Gangwon‐do Republic of Korea

**Keywords:** fibroblast, keloids, radiotherapy, recurrence, single fraction, vitamin D

## Abstract

**Background:**

Keloids, characterized by excessive collagen deposition, often recur despite various treatments. This study explores the association between preoperative serum vitamin D levels and keloid recurrence in a Korean population.

**Methods:**

A retrospective cohort of 160 patients who underwent keloid excision was analyzed. Preoperative serum 25(OH) vitamin D and 1,25(OH)_2_ vitamin D levels were measured. Recurrence rates were compared using hierarchical logistic regression, adjusting for potential confounders.

**Results:**

Age was significantly associated with keloid recurrence (OR: 0.934, *p* = 0.009), indicating older age was linked to lower recurrence risk. No significant association was found between preoperative 25(OH) vitamin D (*p* = 0.395) and 1,25(OH)_2_ vitamin D levels (*p* = 0.925) and keloid recurrence.

**Conclusions:**

Preoperative vitamin D levels do not predict keloid recurrence in this Korean cohort, while age is a significant predictor. Understanding the multifactorial nature of keloid pathogenesis requires further investigation into other potential risk factors.

## 1 Introduction

Scars have the strange power to remind us that our past is real. Cormac McCarthy [1].

This evocative quote by the acclaimed author Cormac McCarthy encapsulates the essence of keloids, pathological scars that serve as tangible reminders of past wounds, both physical and emotional. Keloids are characterized by excessive collagen deposition and growth beyond the original wound boundaries, arising from an aberrant wound‐healing process [2, 3]. These scars not only cause cosmetic disfigurement but also lead to physical discomfort, functional impairment, and psychological distress, thereby significantly impacting patients' quality of life [4].

The presence of keloids serves as a constant reminder of the trauma endured, etching the past into the present. Despite the availability of various treatment modalities, such as surgical excision, intralesional corticosteroids, and radiotherapy, keloid recurrence rates remain high [5]. The persistent nature of keloids and the lack of universally effective treatments highlight the urgent need to better understand their complex pathogenesis and identify novel risk factors and therapeutic targets.

Recent studies have suggested a potential link between vitamin D, a crucial regulator of various biological processes, and keloid formation [6, 7, 8]. Lower serum vitamin D levels have been observed in keloid patients compared to healthy controls, indicating a possible association between vitamin D deficiency and keloid susceptibility [6]. In vitro studies have demonstrated that vitamin D can modulate the biological activities of keloid fibroblasts, the primary effector cells in keloid pathogenesis, by inhibiting their proliferation and migration while promoting apoptosis and reducing the expression of profibrotic factors [9, 10].

However, the specific relationship between vitamin D status and keloid recurrence, particularly in Asian populations who are more prone to both keloids and vitamin D deficiency, remains largely unexplored. Most existing studies have employed cross‐sectional designs with small sample sizes, primarily focusing on the association between vitamin D status and keloid occurrence rather than recurrence [7]. To address this gap in knowledge, we conducted a retrospective study to investigate the association between preoperative serum vitamin D levels and keloid recurrence in a Korean population. We hypothesized that lower preoperative vitamin D levels would be associated with a higher risk of keloid recurrence.

To the best of our knowledge, this is the first study to specifically examine this relationship in an Asian population, considering a wide range of patient, keloid, and treatment characteristics. By elucidating the link between vitamin D status and keloid recurrence while controlling for potential confounders, we aimed to provide valuable insights into the role of vitamin D in keloid pathogenesis and its potential implications for risk stratification and targeted interventions. The multifactorial nature of keloid pathogenesis, involving a complex interplay of genetic predisposition, local skin factors, and systemic influences, underscores the importance of a comprehensive approach to understanding the mechanisms underlying keloid formation and recurrence [11].

Through this research, we hope to contribute to the ongoing efforts to unravel the intricate puzzle of keloid pathogenesis and to develop more effective strategies for their prevention and treatment. By deepening our understanding of the role of vitamin D in the context of keloid recurrence, we may uncover new avenues for risk assessment, targeted therapies, and personalized management approaches. Ultimately, our goal is to alleviate the suffering caused by these enigmatic scars and to help patients move beyond the shadows of their past wounds, both physically and emotionally.

## 2 Patients and Methods

### 2.1 Ethical Considerations

The study adhered to the ethical standards of the institutional or national research committee and the 1964 Helsinki Declaration and its later amendments or comparable ethical standards. The study protocol was approved by the institutional review board.

### 2.2 Study Design and Population

**FIGURE 1:**
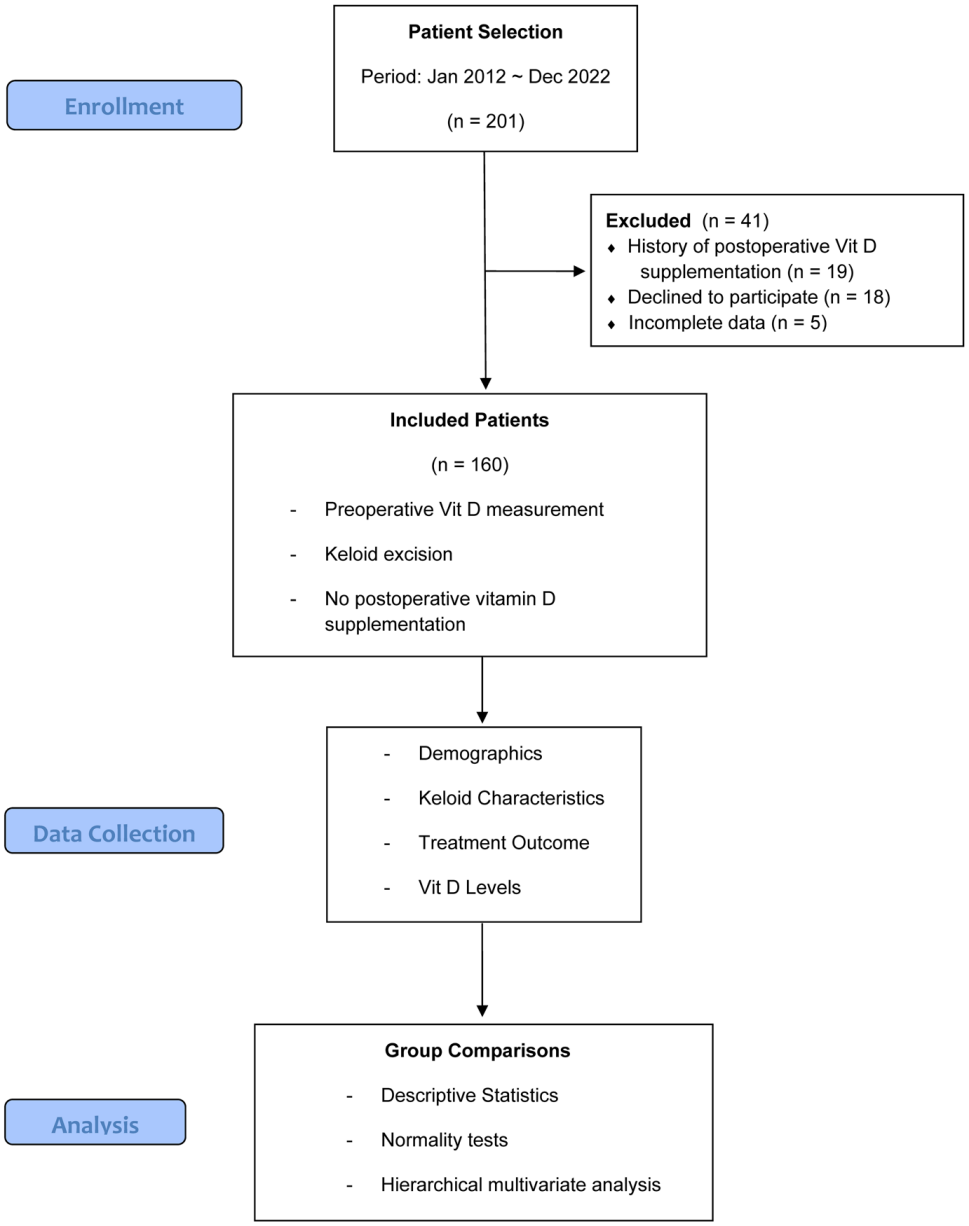
Study design and patient selection flow chart.

This retrospective study was conducted at a tertiary medical center in the Republic of Korea. The medical records of 201 patients who underwent surgical excision for keloids between January 2012 and December 2022 were reviewed. Recurrence was assessed during regular follow‐up visits at 3‐month intervals for the first year and 6‐month intervals thereafter, with a minimum follow‐up period of 1 year. Recurrence was defined as any new keloid formation at the surgical site, confirmed by clinical examination. For patients who did not receive radiotherapy, alternative treatments such as intralesional triamcinolone injection (20–40 mg/mL) or silicone‐based scar gel were provided based on individual clinical needs. The study cohort included male and female patients of various ages, all of whom provided informed consent for the use of their medical data in research. Patients were included in the study if they had preoperative vitamin D measurement, underwent keloid excision, and did not take postoperative vitamin D supplements. A total of 160 patients were included in the final analysis, while 41 patients were excluded due to postoperative vitamin D supplementation, declined participation, or incomplete data (Figure 1).

### 2.3 Rationale for Exclusion of Vitamin D Supplementation

Patients who took vitamin D supplements post‐operatively were excluded from the study for the following reasons:

1. Avoiding Confounding Effects: Post‐operative vitamin D supplementation could lead to significant fluctuations in serum vitamin D levels, potentially confounding the analysis of the relationship between preoperative vitamin D levels and keloid recurrence.
2. Maintaining Study Integrity: Including both supplemented and non‐supplemented patients could introduce variability that undermines the reliability and consistency of the study results.

The exclusion was based solely on patients' voluntary decision to take supplements and not due to any intervention by the researchers. All patients received standard care according to their individual needs, regardless of study participation.

### 2.4 Rationale for Pre‐Operative Only Vitamin D Measurement

The study design included only pre‐operative vitamin D measurements for several methodological considerations:

1. Surgical Impact on Metabolism
  - Surgical trauma and wound‐healing processes significantly alter systemic vitamin D metabolism [12].
  - Complex tissue responses and inflammatory mediators directly influence vitamin D binding protein levels [13, 14].
2. Baseline Status as Predictor
  - Pre‐operative vitamin D status provides a more stable indicator for investigating its relationship with keloid recurrence [6].
  - Post‐operative measurements would reflect a combination of pre‐existing status and surgery‐induced changes.
  - The retrospective nature of the study limited standardized post‐operative measurements.
3. Clinical Practice:
  - Post‐operative vitamin D measurements are not part of standard clinical care [15].
  - Additional blood sampling solely for research purposes raises ethical concerns in retrospective studies.

### 2.5 Data Collection

Data collected for each patient included:

1. Demographic Information: Age and gender
2. Keloid Characteristics: Anatomical location and number removed of keloids
3. Treatment Information: Use of radiotherapy (yes/no) and timing of radiotherapy post‐operatively (post‐operative day 0/1)
4. Vitamin D Levels: Preoperative serum levels of 25(OH) vitamin D and 1,25(OH)_2_ vitamin D
5. Outcome: Keloid recurrence (yes/no)

To minimize the effects of diurnal variation, blood samples for vitamin D measurement were collected at a standardized time (9–10 a.m.) for all included patients.

### 2.6 Statistical Analysis

#### 2.6.1 Descriptive Statistics

Continuous variables were summarized as mean, standard deviation (SD), median, and interquartile range (IQR). Categorical variables were summarized as frequencies and percentages.

#### 2.6.2 Normality Tests

To evaluate the distribution of continuous variables (age, 25(OH) vitamin D level, and 1,25(OH)_2_ vitamin D level) in both recurrence and non‐recurrence groups, Shapiro–Wilk tests were conducted to assess normality.

#### 2.6.3 Group Comparisons

Given that none of the continuous variables satisfied the normality assumption (as determined by the Shapiro–Wilk tests), non‐parametric methods were employed for group comparisons. Specifically, the Mann–Whitney *U* test was used to compare differences between the recurrence and non‐recurrence groups for age, 25(OH) vitamin D level, and 1,25(OH)_2_ vitamin D level.

#### 2.6.4 Multivariate Analysis

Hierarchical logistic regression analysis was performed to assess the relationship between vitamin D levels and keloid recurrence while adjusting for potential confounders (age, gender, anatomical location, use of radiotherapy). Results were reported as odds ratios (OR) with 95% confidence intervals (CI) and *p* values.

#### 2.6.5 Subgroup Analyses

The impact of radiotherapy on keloid recurrence was evaluated by comparing recurrence rates among patients who received radiotherapy on postoperative day 0, postoperative day 1, and those who did not receive radiotherapy. The Fisher–Freeman–Halton exact test was employed due to the small expected frequencies in some cells, which made it a suitable method for this 2 × 3 group comparison. Additionally, differences based on the anatomical locations of keloids were analyzed.

#### 2.6.6 Power Analysis

A post hoc power analysis was conducted to determine the study's ability to detect a significant effect of vitamin D levels on keloid recurrence. The power analysis was performed using the observed effect sizes and standard deviations with an alpha level of 0.05.

## 3 Results

### 3.1 Patient Demographics and Baseline Characteristics

The study cohort comprised 160 patients with a mean age of 34.39 years. 110 female patients (68.8%) and 50 male patients (31.2%). While the mean number of excised keloids per patient was 1.30 ± 0.69, this narrow range of variation limited our statistical analysis of the relationship between keloid number and recurrence. Keloid locations were distributed as follows: ear (43.8%), anterior chest (23.8%), head and neck (6.9%), and other locations. Radiotherapy was administered to 108 patients (67.5%), with a majority receiving a dosage of 10 Gy on the day of surgery (52.5%; Table 1).

**TABLE 1:** Patient demographics between two groups.

Characteristic	Total (*n* = 160)	No recurrence (*n* = 142)	Recurrence (*n* = 18)	*p*
Age (years) ± SD	34.39 ± 14.37	35.42 ± 14.31	26.28 ± 12.30	0.006
Gender				0.458
Male	50 (31.25%)	43 (30.28%)	7 (38.89)	
Female	110 (68.75%)	99 (69.72%)	11 (61.11%)	
Lesion location				
Head and neck	11 (6.88%)	7 (4.93%)	4 (22.22%)	0.023
Ear	70 (43.75%)	66 (46.48%)	4 (22.22%)	0.051
Anterior chest	38 (23.75%)	31 (21.83%)	7 (38.89)	0.140
Other	41 (25.62%)	38 (26.76%)	3 (16.66%)	
Number of lesions removed ± SD	1.30 ± 0.69	1.28 ± 0.63	1.47 ± 1.01	0.423
Postoperative radiotherapy	108 (67.50%)	95 (66.90%)	13 (72.22%)	0.142
POD #0	84 (52.5%)	71	13	
POD #1	24 (15%)	24	0	

*Note:* Continuous variables (age, number of lesions removed) are summarized as mean ± standard deviation (SD). For normally distributed variables, group differences were compared using the *t*‐test. For non‐normally distributed variables, the Mann–Whitney *U* test was used. Categorical variables (sex, lesion location, postoperative radiotherapy) are summarized as frequencies and percentages, and compared using the chi‐square test or Fisher's exact test where appropriate. Significant differences are indicated by *p* < 0.05.

### 3.2 Vitamin D Levels and Keloid Recurrence

**FIGURE 2:**
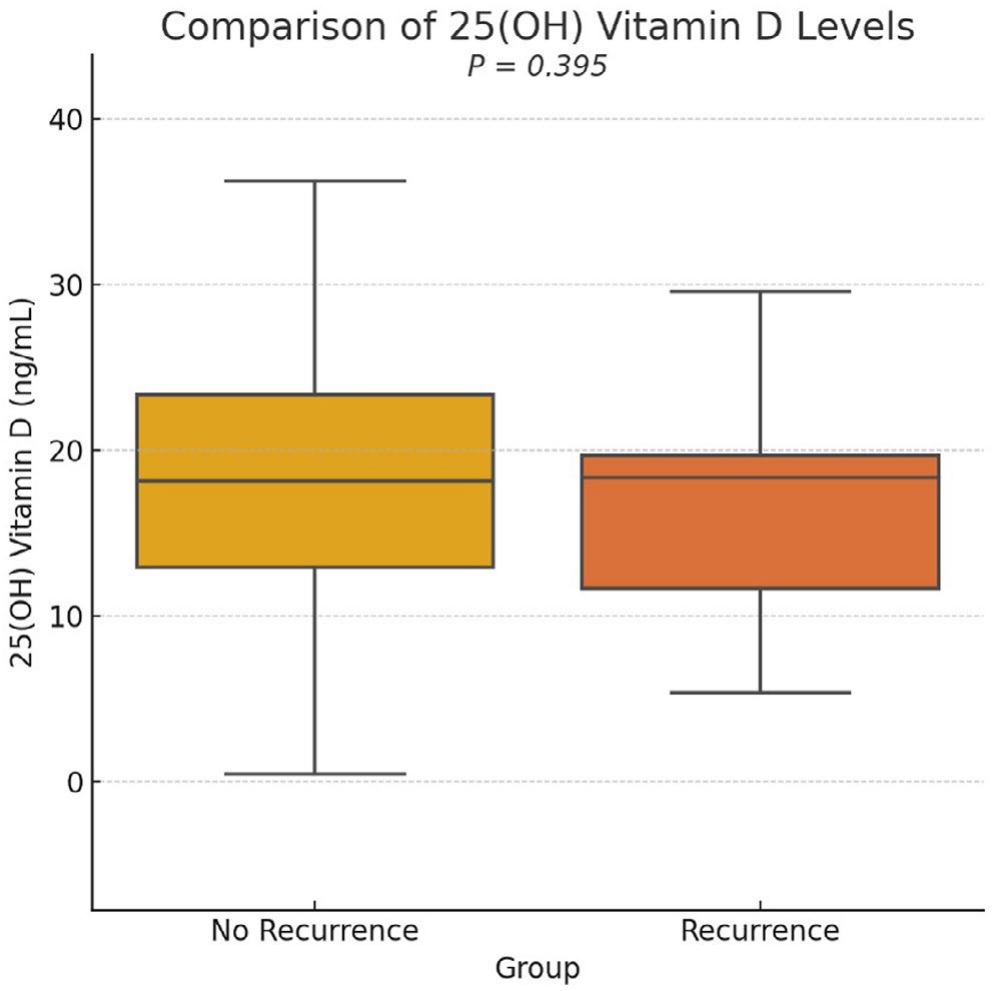
Comparison of 25(OH) vitamin D levels. Boxplot comparing the 25(OH) vitamin D levels between Keloid recurrence and non‐recurrence groups. Statistical analysis was performed using the Mann–Whitney *U* test. *p* = 0.395 indicates no significant difference.

**FIGURE 3:**
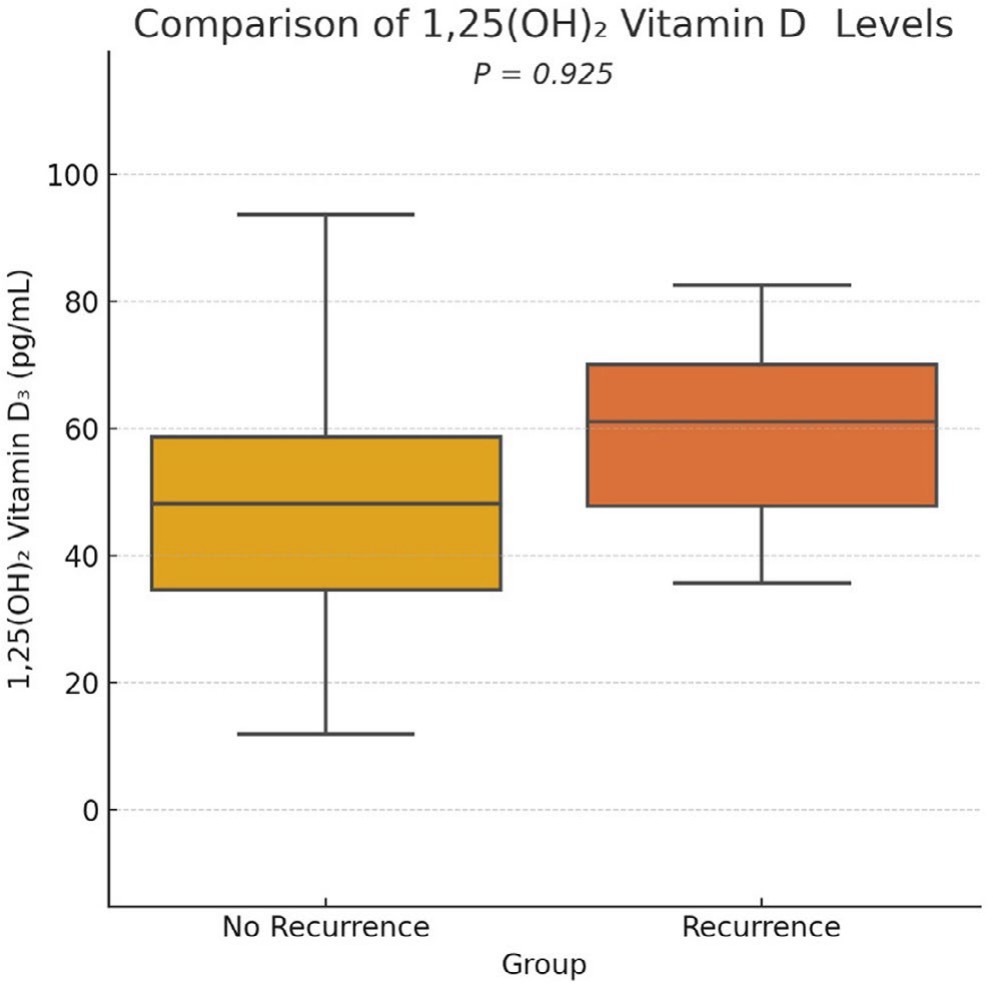
Comparison of 1,25(OH)_2_ vitamin D levels. Boxplot comparing the 1,25(OH)_2_ Vitamin D3 levels between keloid recurrence and non‐recurrence groups. Statistical analysis was performed using the Mann–Whitney *U* test. *p* = 0.925 indicates no significant difference.

The median serum 25(OH) vitamin D level was 16.3 ng/mL (IQR: 11.8–23.1) in the no recurrence group and 15.7 ng/mL (IQR: 10.2–20.1) in the recurrence group, with no statistically significant difference (*p* = 0.395; Figure 2). The median serum 1,25(OH)_2_ vitamin D level was 43.9 pg/mL (IQR: 34.4–58.9) in the no recurrence group and 39.4 pg/mL (IQR: 32.5–68.0) in the recurrence group (Table 2), also with no statistically significant difference (*p* = 0.925) (Figure 3).

**TABLE 2:** Vitamin D levels in keloid recurrence and non‐recurrence groups.

Variable	Total (*n* = 160)	No recurrence (*n* = 142)	Recurrence (*n* = 18)	*p*	Odds ratio (95% CI)
25(OH) vitamin D					
Mean ± SD (ng/mL)	18.57 ± 9.02	18.83 ± 9.24	16.59 ± 6.98	—	—
Median (IQR) (ng/mL)	16.1 (11.8–23.1)	16.3 (11.8–23.1)	15.7 (10.2–20.1)	0.395	0.968 (0.908–1.032)
1,25(OH)_2_ vitamin D3					
Mean ± SD (pg/mL)	47.23 ± 18.18	46.84 ± 17.25	50.31 ± 24.75	—	—
Median (IQR) (pg/mL)	43.8 (34.4–59.5)	43.9 (34.4–58.9)	39.4 (32.5–68.0)	0.925	1.010 (0.984–1.038)

*Note:* The Mann–Whitney *U* test was conducted to compare the median levels of 25(OH) Vitamin D and 1,25(OH)_2_ Vitamin D3 between the keloid recurrence group and the non‐recurrence group. The statistical analysis showed no significant differences in vitamin D levels.

### 3.3 Hierarchical Logistic Regression Analysis

A stepwise hierarchical logistic regression analysis was conducted to assess the relationship between vitamin D levels and keloid recurrence while adjusting for potential confounders. The analysis was performed in four steps:

1. Basic Demographic Variables: Age and gender were included in the first step. The results showed that age was significantly associated with keloid recurrence (OR: 0.934, 95% CI: 0.887–0.983, *p* = 0.009), indicating that older age was associated with a lower risk of recurrence. Gender did not show a significant association (OR: 0.476, 95% CI: 0.161–1.403, *p* = 0.178).
2. Keloid Characteristics: Anatomical location and number of keloids were added in the second step. Neither anatomical location (OR: 0.989, 95% CI: 0.585–1.675, *p* = 0.968) nor number of keloids (OR: 1.162, 95% CI: 0.617–2.187, *p* = 0.632) were significantly associated with recurrence.
3. Treatment Information: Use of radiotherapy and timing of radiotherapy were included in the third step. Use of radiotherapy was not significantly associated with recurrence (OR: 1.20, 95% CI: 0.047–30.907, *p* = 0.912). Timing (OR: 0.715, 95% CI: 0.140–3.657, *p* = 0.687) also did not show significant associations.
4. Vitamin D Levels: Finally, 25(OH) vitamin D and 1,25(OH)_2_ vitamin D levels were added. The levels of 25(OH) vitamin D (OR: 0.993, 95% CI: 0.919–1.074, *p* = 0.866) and 1,25(OH)_2_ vitamin D (OR: 1.011, 95% CI: 0.982–1.041, *p* = 0.464) were not significantly associated with keloid recurrence (Table 3).

**TABLE 3:** Hierarchical logistic regression analysis results.

Variables	Odds ratio (OR)	95% confidence interval (CI)	*p*
Step 1: Basic demographic variables			
Age	0.934	0.887–0.983	0.009
Gender	0.476	0.161–1.403	0.178
Step 2: Adding keloid characteristics			
Age	0.935	0.887–0.985	0.012
Gender	0.504	0.165–1.545	0.231
Anatomical location	0.989	0.585–1.675	0.968
Number of removed keloids	1.162	0.617–2.187	0.642
Step 3: Adding treatment information			
Age	0.923	0.870–0.980	0.008
Gender	0.584	0.186–1.831	0.356
Anatomical location	0.987	0.596–1.634	0.958
Number of removed keloids	1.060	0.556–2.021	0.860
Use of Rx.	1.201	0.047–30.907	0.912
Timing of Rx.	0.715	0.140–3.657	0.687
Step 4: Adding vitamin D levels			
Age	0.929	0.874–0.987	0.017
Gender	0.538	0.165–1.758	0.305
Anatomical location	0.992	0.597–1.649	0.975
Number of removed keloids	1.086	0.565–2.087	0.804
Use of RTx.	0.846	0.029–24.808	0.923
Timing of RTx.	0.600	0.110–3.292	0.557
25(OH) Vit D	0.993	0.919–1.074	0.866
1,25(OH)_2_ Vit D	1.011	0.982–1.041	0.464

*Note:* The hierarchical logistic regression analysis was performed in four steps. The table presents the odds ratios (OR), 95% confidence intervals (CI), and *p* values for each variable included in the analysis. Only age showed statistical significance.

Abbreviation: RTx., radiotherapy.

### 3.4 Subgroup Analyses

The impact of radiotherapy on keloid recurrence was evaluated. Patients who received radiotherapy had a recurrence rate of 12.04% compared to 9.62% in those who did not, although this difference was not statistically significant (*p* = 0.792; Table 4). The association between the timing of radiotherapy and keloid recurrence was analyzed using the Fisher–Freeman–Halton exact test, as the expected frequencies in some cells were less than 5. The test revealed no statistically significant difference in recurrence rates based on the timing of radiotherapy (*p* = 0.152).

**TABLE 4:** Impact of radiotherapy on keloid recurrence.

	Total	No recurrence	Recurrence	Recurrence rate (%)	*p*
Radiotherapy (yes)	108	95	13	12.04	0.792 (Fisher's exact)
Radiotherapy (no)	52	47	5	9.62	
Timing of radiotherapy					0.152 (Fisher–Freeman–Halton exact)
POD #0	84	71	13	14.46	
POD #1	24	24	0	0	

*Note: p* < 0.05 was considered statistically significant.

### 3.5 Post Hoc Power Analysis

A post hoc power analysis was conducted to evaluate the study's ability to detect a significant effect of vitamin D levels on keloid recurrence. Based on the observed effect size of 0.1 (small effect size), an alpha level of 0.05, and a sample size of 160 patients, the power analysis indicated that the study had a power of approximately 14.5% to detect a significant difference.

## 4 Discussion

This retrospective study investigated the association between preoperative serum vitamin D levels and keloid recurrence in a Korean population. Despite the biological plausibility of vitamin D playing a role in skin health and wound healing, our findings did not establish a significant relationship between serum Vitamin D levels and the risk of keloid recurrence after adjusting for potential confounders such as age, gender, lesion location, and radiotherapy use.

The lack of association between vitamin D levels and keloid recurrence in this study could be attributed to several factors. First, the pathogenesis of keloids is multifactorial, involving complex interactions between genetic predisposition, local skin conditions, and systemic factors [16]. Vitamin D's role might be overshadowed by more dominant factors influencing keloid recurrence, such as local mechanical stress and inflammation. Furthermore, the biological activity of vitamin D is mediated through its receptor, VDR, which is expressed in various skin cells [17]. Studies have shown that VDR expression is downregulated in keloid fibroblasts compared to normal skin fibroblasts [18], suggesting an impaired response to vitamin D in keloid tissues. This local resistance to vitamin D action might contribute to the persistence and recurrence of keloids despite adequate or supplemented serum vitamin D levels.

### 4.1 Key Findings and Interpretations

The results indicated that the median serum 25(OH) vitamin D levels were comparable between the non‐recurrence group (16.3 ng/mL) and the recurrence group (15.7 ng/mL; *p* = 0.395). Similarly, the median serum 1,25(OH)_2_ vitamin D levels did not differ significantly between the non‐recurrence group (43.9 pg/mL) and the recurrence group (39.4 pg/mL; *p* = 0.925). These findings suggest that while vitamin D deficiency may be prevalent among keloid patients, as corroborated by previous studies [6, 7, 9, 18], its direct impact on keloid recurrence might be limited. The lack of association between Vitamin D levels and keloid recurrence could be attributed to the complex interplay of factors involved in keloid pathogenesis, with vitamin D playing a less dominant role compared to other local and systemic factors.

Additionally, genetic variations in the VDR gene may influence individual responses to vitamin D, further complicating the relationship between serum vitamin D levels and keloid recurrence. The expression and activity of VDR in keloid tissues may be more relevant to the local effects of vitamin D on keloid formation and recurrence than serum levels alone [19].

Interestingly, this study identified patient age as a significant predictor of keloid recurrence, with younger patients exhibiting a higher risk. This finding aligns with the literature and could be explained by age‐dependent differences in wound‐healing processes, inflammatory responses, and collagen metabolism [20, 21]. Younger individuals typically exhibit a more robust inflammatory response and higher collagen synthesis capacity [22], which may predispose them to excessive scar formation and keloid recurrence.

Although the distribution of keloid location in the head and neck region showed a statistically significant difference between the recurrence and non‐recurrence groups using the Mann–Whitney *U*‐test (*p* = 0.023), this factor was not significant in the multivariate regression analysis. This suggests that while anatomical location may play a role in keloid formation [23], other factors may have a more dominant influence on recurrence risk. Areas with higher skin tension, such as the head and neck, are more prone to abnormal wound healing due to repetitive mechanical stress [24]. Moreover, these regions are more exposed to ultraviolet [25, 26], which can induce skin aging and inflammation, further promoting keloid formation and recurrence [27, 28]. The unique anatomical and physiological properties of these sites, including thinner epidermis and higher density of hair follicles, may render them more vulnerable to keloid development. However, these factors did not independently predict recurrence in the regression model, indicating the need for further research to elucidate their role.

### 4.2 Strengths and Limitations

Our study's strengths include a relatively large sample size from a single institution, which minimized potential confounding due to variations in treatment protocols and follow‐up procedures. Comprehensive data collection allowed for controlling key confounding variables, and rigorous statistical analyses were performed to validate our findings.

However, several limitations must be acknowledged. The retrospective design may introduce selection and information biases. The small number of recurrence events (*n* = 18) limited our statistical power, as evidenced by the low power (14.5%) in the post hoc analysis. This small sample size in the recurrence group may have hindered the detection of statistically significant associations. The single‐center setting and predominantly Korean population may limit the generalizability of the above findings to other ethnic groups and healthcare contexts. Additionally, the single time point measurement of preoperative vitamin D levels may only partially capture its relationship with keloid recurrence. While post‐operative vitamin D measurements could provide additional insights, these were not included as surgical trauma and wound‐healing processes significantly alter systemic vitamin D metabolism and local tissue responses [14], making it challenging to interpret the direct relationship between vitamin D levels and keloid recurrence in the post‐operative period [13]. Future prospective studies should consider systematic monitoring of vitamin D levels at multiple time points and investigate the impact of vitamin D supplementation, along with evaluation of local vitamin D receptor expression in keloid tissues, to better understand this complex relationship.

Another limitation was the narrow distribution of excised keloid numbers (1.30 ± 0.69) in our study population. This uniform distribution, while reflecting the common clinical presentation at our institution, restricted our ability to fully investigate whether multiple keloids could be a risk factor for recurrence. Previous studies have suggested that patients with multiple keloids might have different biological characteristics or stronger genetic predisposition to keloid formation, potentially affecting their recurrence patterns [29]. However, our cohort's relatively homogeneous presentation of predominantly single or double keloids prevented us from exploring this relationship. Future studies incorporating patients with a wider range of keloid numbers would be valuable in determining whether the number of keloids could serve as a predictor of recurrence risk.

### 4.3 Future Directions and Implications

Future studies should adopt prospective designs with larger, diverse cohorts and longer follow‐up periods to better elucidate the role of vitamin D in keloid pathogenesis and recurrence. Investigating the local expression and activity of VDR in keloid tissues, as well as the potential impact of VDR polymorphisms, could provide more concrete insights into the mechanisms by which vitamin D influences keloid formation. Additionally, examining other relevant biomarkers, such as inflammatory cytokines, growth factors, and matrix metalloproteinases, may help clarify the specific biological pathways involved.

## 5 Conclusion

This study found no significant association between preoperative serum vitamin D levels and keloid recurrence in a Korean population. However, patient age emerged as significant predictors of recurrence risk, highlighting keloid pathogenesis's complex and multifactorial nature. While the findings of this study provide valuable insights into the role of vitamin D in keloid recurrence, they should be interpreted with caution, considering the limitations of the study design and sample size. Future research employing a multidimensional approach to investigate the interplay between systemic and local vitamin D status, genetic variations, and other potential risk factors is crucial for advancing our understanding of keloid pathogenesis and developing targeted prevention and treatment strategies. By mastering the intricate dynamics of keloid formation and recurrence, we can better arm ourselves in the battle against this persistent adversary, ultimately enhancing the quality of life for those affected.

## Conflicts of Interest

The authors declare no conflicts of interest.

## Data Availability

The data that support the findings of this study are available from the corresponding author upon reasonable request.
